# Deep learning workflow in radiology: a primer

**DOI:** 10.1186/s13244-019-0832-5

**Published:** 2020-02-10

**Authors:** Emmanuel Montagnon, Milena Cerny, Alexandre Cadrin-Chênevert, Vincent Hamilton, Thomas Derennes, André Ilinca, Franck Vandenbroucke-Menu, Simon Turcotte, Samuel Kadoury, An Tang

**Affiliations:** 1 Centre de recherche du Centre Hospitalier de l’Université de Montréal (CRCHUM), Montréal, Québec Canada; 2 Department of Medical Imaging, CISSS Lanaudière, Université Laval, Joliette, Québec Canada; 3 Department of Radiology, Radio-Oncology and Nuclear Medicine, Université Montréal and CRCHUM, 1058 rue Saint-Denis, Montréal, Québec, H2X 3 J4 Canada; 4 Department of Surgery, Hepatopancreatobiliary and Liver Transplantation Service, Centre Hospitalier de l’Université de Montréal (CHUM), Montréal, Quebec Canada; 5 Polytechnique Montréal, Montréal, Québec Canada

**Keywords:** Review article, Deep learning, Medical imaging, Cohorting, Convolutional neural network

## Abstract

Interest for deep learning in radiology has increased tremendously in the past decade due to the high achievable performance for various computer vision tasks such as detection, segmentation, classification, monitoring, and prediction. This article provides step-by-step practical guidance for conducting a project that involves deep learning in radiology, from defining specifications, to deployment and scaling. Specifically, the objectives of this article are to provide an overview of clinical use cases of deep learning, describe the composition of multi-disciplinary team, and summarize current approaches to patient, data, model, and hardware selection. Key ideas will be illustrated by examples from a prototypical project on imaging of colorectal liver metastasis. This article illustrates the workflow for liver lesion detection, segmentation, classification, monitoring, and prediction of tumor recurrence and patient survival. Challenges are discussed, including ethical considerations, cohorting, data collection, anonymization, and availability of expert annotations. The practical guidance may be adapted to any project that requires automated medical image analysis.

## Key points

- Deep learning provides state-of-the-art performance for detection, segmentation, classification, and prediction.
- A multi-disciplinary team with clinical, imaging, and technical expertise is recommended.
- Data collection and curation constitute the most time-consuming steps.
- Several open-source deep learning frameworks with permissive licenses are available.
- Cloud computing leverages third-party hardware, storage, and technical resources.

## Introduction

Deep learning is a subtype of representation learning which aims to describe complex data representations using simpler hierarchized structures defined from a set of specific features. With the advent of powerful parallel computing hardware based on graphical processing units (GPUs) and the availability of large datasets, deep learning has become a state-of-the-art technique in computer vision [1]. In the context of healthcare, deep learning shows great promise for analyzing structured (e.g., databases, tables) and unstructured data (e.g., images, text) [2]. Over the past decade, medical image analysis has greatly benefited from the application of deep learning (DL) techniques to various imaging modalities and organs [3].

Several tasks traditionally performed by radiologists such as lesion detection, segmentation, classification, and monitoring may be automated using deep learning techniques [4]. In abdominal radiology, deep learning has been applied to diverse tasks [3], organs [5, 6], and pathologies [7–9]. Despite the emerging application of deep learning techniques [1, 10], few articles have described the workflow to execute projects in abdominal radiology which require a broad range of steps, ranging from selection of patient population, choice of index test and reference standard, model selection, and assessment of performance.

The purpose of this narrative review is to provide a practical guide for radiologists interested in conducting a project that involves deep learning in abdominal radiology. We will cover each step in the chronological order of a project. Specifically, the objectives of this article are to (1) provide an overview of clinical use cases, (2) describe the composition of multi-disciplinary team, (3) and summarize current approaches to patient, data, model, and hardware selection. We will do so by providing examples from a prototypical project that involves imaging of colorectal liver metastasis. We will illustrate the workflow in the context of liver lesion detection, segmentation, classification, monitoring, and prediction of tumor recurrence and patient survival. While this article is intended for abdominal radiologists, the practical guidance may be adapted to other projects that require automated medical image analysis.

## Overview of project

A checklist of representative steps required for management of a deep learning project is provided in Table 1.

**Table 1:** Checklist of steps required for management of project involving deep learning

Scope	❏ Define scope of project: detection, segmentation, classification, monitoring, prediction or prognosis.
Team building	❏ Project manager (e.g, physician, data scientist) ❏ Clinical expertise (e.g., surgeon or hepatologist) ❏ Imaging expertise (e.g., radiologist) ❏ Technical expertise (e.g., data scientist)
Ethics	❏ Obtain IRB approval
Cohorting	❏ Selection process (e.g., by target population vs. database) ❏ Definition of eligibility criteria ❏ Identification of data source
Data	*De-identification* ❏ Data anonymization vs. pseudonymization *Collection and curation* ❏ Data collection ❏ Data exploration and quality control ❏ Labeling = markup and annotations ❏ Reference standard (synonyms: ground truth or gold standard) *Sampling* ❏ Creation of training, validation and test datasets ❏ Alternative: cross-validation
Model	❏ Defining performance metrics ❏ Selection of model (convolutional, recurrent, fully connected) and librairies ❏ Running the experiment followed by hyperparameters fine tuning ❏ Testing: assessing performance on separate test dataset
Hardware	❏ Determine best configuration based on model architecture and memory requirements ❏ Local (CPUs vs. GPUs) vs. cloud computing (GPUs vs. TPUs)
Regulatory	❏ Market research to inform decision to commercialize ❏ Quality management system ❏ Compliance with local regulatory jurisdictions
Clinical adoption	❏ Integration in distribution platform ❏ Clinical validation of performance ❏ Deployment in clinical practice

## Overview of clinical use of deep learning

**Fig. 1:**
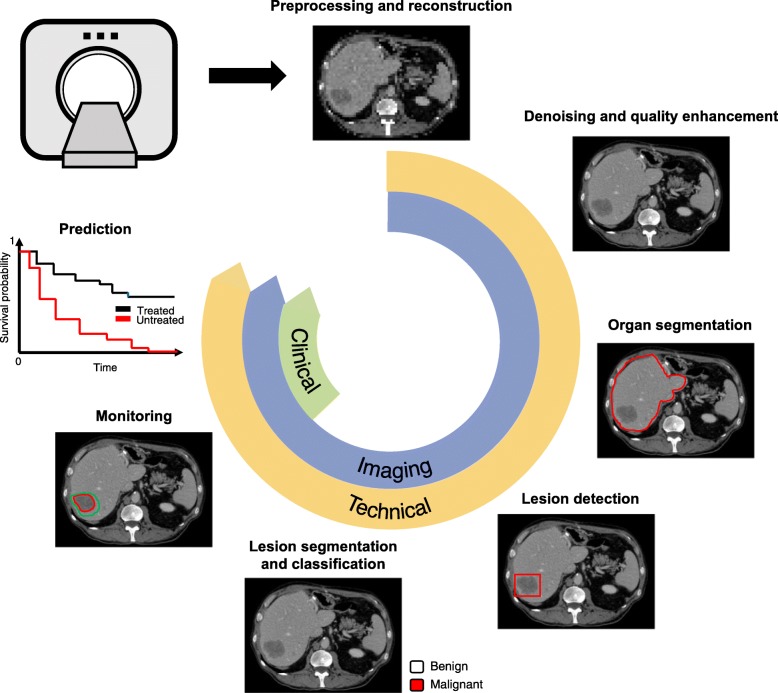
Potential clinical uses of deep learning techniques. Tasks such as monitoring of treatment response or prediction of survival, can be derived from lesion detection, classification, and longitudinal follow-up

Figure 1 illustrates some potential clinical uses of deep learning techniques. Clinical use refers to the range of applications in healthcare context, such as clinical workflow optimization, improved computer-aided diagnosis (CAD), and computer-assisted reporting [11]. Deep learning may be used for automation of various time-consuming tasks performed by radiologists such as lesion detection, segmentation, classification, monitoring, and also prediction of treatment response which is usually not achievable without software. Of note, the distinction between these tasks is conceptual because some algorithms can accomplish several tasks simultaneously (e.g., detection, segmentation, and classification [12]). Furthermore, detection and segmentation are subtypes of classification tasks, since they consist in categorizing image regions or pixels based on a predefined criterion (e.g., tissue or lesion type). While neural networks extract image features through the learning process, the use of quantitative image-based features (e.g., statistics of the intensity distribution, textures), referred as “radiomics” in a machine learning context, has been proposed [13, 14].

- *Types of tasks*

1. Image preprocessing refers to techniques applied either on raw signals or on reconstructed images. For example, deep learning methods have been used for image reconstruction from sparse MRI data [15] or for improving image quality with noise and artifact reduction [16], super resolution and image acquisition and reconstruction [17].
2. *Detection* refers to highlighting a specific subregion in an image which is likely to contain a localized tissue heterogeneity (focal lesion or anomaly). For example, in the presence of liver metastases, the purpose of lesion detection is to roughly identify individual lesions with bounding boxes [5, 18].
3. *Segmentation* refers to delineation or volume extraction of a lesion or organ based on image analysis (e.g., pixel intensity, texture, edges) [19]. For example, in patients with liver metastases, lesion segmentation would outline the contour of metastases to extract the largest diameter in long and short axes for subsequent monitoring of response to chemotherapy [20, 21] or to compute tumor volumetry to estimate the volume of the future liver remnant [22].
4. *Classification* refers to categorization of a specific group or type to a lesion from one class to others. Such classification may be binary (e.g*.*, benign or malignant) or multi-class (various subtypes of lesions). For example, in patients with liver metastases, the purpose of lesion classification is to differentiate benign lesions (such as focal liver fat, cysts, and hemangiomas) from malignant lesions (such as primary or secondary liver cancer) [7].
5. *Monitoring* refers to longitudinal follow-up of a specific *lesion* over time to assess changes in appearance, diameter, or volume. For example, in patients with liver metastases, the purpose of lesion monitoring is to assess disease progression, stability, or regression over time [23]. In order to quantify the evolution of focal disease, segmentation of focal lesions and the corresponding organ is required to assess the percentage of organ affected by lesions [24].
6. *Prediction* refers to leveraging specific features to anticipate the evolution of a pathology. For example, in patients with liver metastases, this task may include prediction of response to chemotherapy, prognosis of recurrence-free disease in treated patients, or overall survival.

## Multi-disciplinary team building

**Fig. 2:**
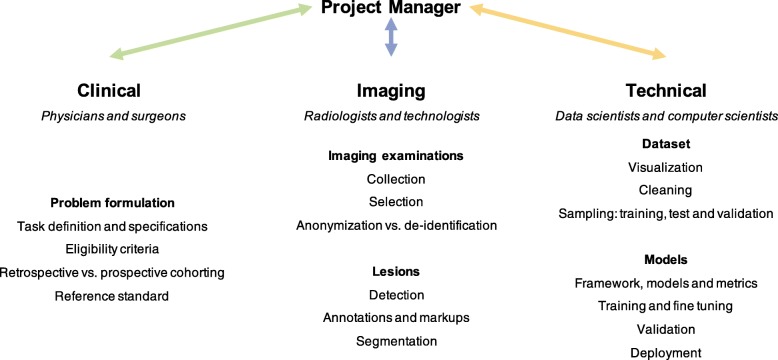
Expertise of team members

Figure 2 illustrates an example of multi-disciplinary expertise and collaboration.

Multi-disciplinary team building refers to a process where people from different fields and levels of expertise are gathered to share their knowledge and collaborate on a joint project. Members are chosen based on the specific needs of the project, such as clinical expertise (e.g., surgeon or hepatologist), imaging expertise (e.g., radiologist), or technical expertise (e.g., data scientist, computer scientist) [25]. Due to the accruing levels of specialization and complexity in healthcare, multi-disciplinary collaborations are expanding. A project manager is required to supervise, coordinate, and maintain communication between team members in order to ensure synchronous work and project flow.

For example, *clinical expertise* (e.g., surgeon or liver oncologists) is required to recruit patients, enrollment in a biobank, identify eligibility for participation in studies, assessment of tumor response grade (TRG), and collect clinical data on type and duration of chemotherapy, details of surgery, time to recurrence, and survival data [26]. *Imaging expertise* (e.g., radiologist and technologists) is required for selection of appropriate imaging examinations, sequences or vascular phases, lesion detection, annotations (e.g., arrows, measurements), segmentation, and classification (e.g., colorectal metastases, cysts, hemangiomas). *Technical expertise* (e.g., data scientist, computer scientist) is required for data anonymization; data cleaning and visualization; creation and splitting of dataset into training, validation, and test datasets; selection of model and libraries; develop and fine-tune the model; validate the performance on a separate test set; and deploy the model.

## Institutional approval

Data collection refers to the process of gathering information from one or more sources for predefined variables to test research hypotheses and assess outcomes [27, 28]. It is a prerequisite for training of deep learning models.

If a project relies on second use of imaging data, approval by institutional review boards must comply with regional regulations such as Health Insurance Portability and Accountability Act in the USA [29], the General Data Protection Regulation in Europe [30], and the Ethical Conduct for Research Involving Humans in Canada [31]. Institutional review boards must enforce the respect of patient autonomy (free, informed and ongoing consent) or waive the need for patient consent (discussed below) and find a balance between risks (e.g., preventing large-scale data breach and unintended disclosure) and benefits (e.g., improving diagnosis and improving treatment selection) [32].

If a study requires tissue biobanking as the reference standard, registration in an online repository such as the Texas Cancer Research Biobank (USA) [33], Manchester Cancer Research Centre (UK) [34], or Cancer Research Network (Canada) [35] may be required.

For prospective studies, informed written consent must usually be obtained prior to enrollment. For retrospective studies, the institutional review board must provide a consent waiver when obtaining explicit consent is impractical, risks associated with data sharing are minimal, and data custodians can be trusted [36].

Data recorded by the biobank include clinical data and biological data, as examination reports, blood or tissue samples. All data in the biobank are anonymized with a key detained only by the biobank manager, and a new identifier is assigned to each patient [37]. The use of collected data is strictly restrained to scientific purposes.

However, the results obtained may contribute to the development of commercial products. Patients can withdraw their consent at any time with destruction of all personal data in the biobank [38].

## Population cohorting

**Fig. 3:**
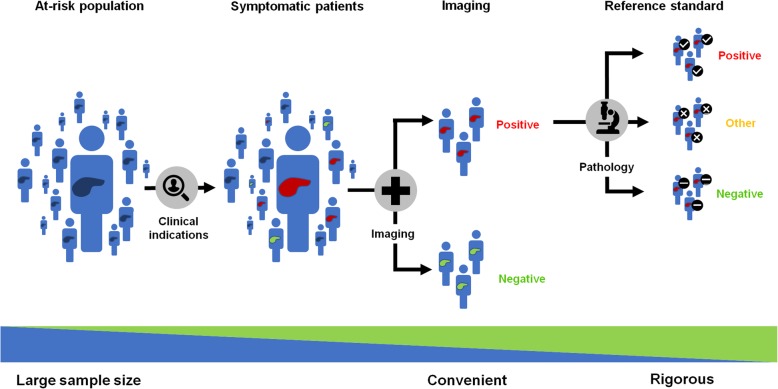
Concept of case selection based on clinical indication (left), imaging (middle), or pathology (right) findings

Figure 3 illustrates the concept of case selection based on clinical criteria (e.g., risk factors or symptoms), imaging examinations, or pathology findings.

Cohorting refers to the identification of patients that share one or more common characteristics, such as patient characteristics (e.g., age, gender), disease characteristics (e.g., disease stage, treatment status), index tests (e.g., ultrasound, computed tomography or MRI), or reference standard (e.g., results of diagnostic imaging test or pathology).

Cohorting may be performed by one of the two following approaches:

1. *a priori* definition of eligibility criteria: with this approach, the inclusion and exclusion criteria may require the availability of any or all of the following: clinical, imaging, or pathological tests.
2. *a posteriori* definition of eligibility criteria: with this approach, the inclusion and exclusion criteria are determined by the available data existing in the repository within a given time interval.

There are trade-offs associated with each patient selection approach.

- *Clinical criteria*: Selecting a study cohort on the basis of clinical legibility criteria provides a large sample size. However, the reference standard may not be available for all patients included (confirmation bias) or may differ between patients with positive findings who may undergo surgery and negative findings who may be followed with imaging (verification bias).
- *Imaging findings*: Selecting a study cohort on the basis of imaging studies is convenient because the index test is available for all included patients. It provides a reasonable trade-off in terms of sample size. However, patients with missing (unavailable examinations from other centers) or inadequate examinations (poor image quality, unenhanced, artifacts) must be excluded.
- *Pathological findings*: Selecting a study cohort on the basis of available tissues specimens and histopathology interpretation provides a rigorous ground truth according to the clinical standard of care. Yet, pathological findings are based on sampling of the surgical specimen (which may not be representative of the entire lesion) and are also subject to interreader variability. Also, requiring pathological findings for all patients included in a cohort limits the sample size to those who have been biopsied or operated.

Depending on the task to be performed (e.g., detection, segmentation, classification, monitoring, prediction or prognosis), the preferred strategy for cohorting may differ. For example, if the aim of a study is to predict the tumor stage, availability of tissue specimens with appropriate treatment response grade scores may be required for cohorting. Subsequently, retrospective retrieval of imaging examinations that will be required to serve as the index test.

## Data de-identification

Practices ensuring privacy of patient-related information are of paramount importance for deep learning projects because sensitive medical information may be reidentified. Thus, three concepts must be kept in mind throughout project planning and execution: de-identification, anonymization, and pseudonymization.

De-identification refers to the masking of patient-related information from individual records in order to minimize the risks of identification and breach of privacy [39].

Anonymization, a subtype of de-identification, refers to the irreversible removal of patient-related information from individual records. It is the preferred approach for sharing of medical data.

Pseudonymization, a subtype of de-identification, refers to the substitution of patient-related information with artificial values in a way that the original data can only be revealed with a secret key [40]. This approach is often required to link different databases. Also, pseudonymization may be required to reidentify patients in case of incidental findings in a clinical research setting. Multiple approaches have been proposed involving variable degrees of encryption from encryption to anonymization [40]. The encryption key should be kept secure, under the responsibility of the principal investigator, and its utilization should be documented [39, 41].

Together with pixel information, digital imaging and communications in medicine (DICOM) files contain additional information that needs to be anonymized in accordance with protected health information regulations [42]. Each type of information is inscribed within one of hundreds of specific, standardly tagged data elements [3]. DICOM headers that can be used to retrieve a patient’s identity, either directly (e.g., name, ID, address) or indirectly (e.g., age, acquisition date, operator) must be anonymized. Supplement 142 of the DICOM Standards provides guidelines regarding the file fields requiring anonymization as well as context-specific recommendations. Free DICOM anonymization softwares are available but should be used with caution, as only a fraction achieves complete data removal, and often, only after thorough customization [43]. DICOM Library [44] and the RSNA Clinical Trials Processor provide two free, proven toolkits for this purpose [45].

## Data collection and curation

Data *collection* refers to aggregation of data, whereas data *curation* refers to exploring and cleaning of data. These steps are performed to standardize and improve dataset quality for subsequent deep neural networks training. Data can be clinical data (biobank), images, and related metadata (DICOM), or annotations (radiology reports). The latter represent human annotations and machine-generated features [46]. This process is typically the most time-consuming step in an AI project, but is critical to any model training. Recently, some general guidelines have been proposed to achieve and maintain high-quality standards in datasets building [14, 47]. While efforts were made to develop automatic curating tools [48], this step still requires human knowledge and supervision to achieve high-quality datasets.

For example, after the selection of eligible cases from a cohort based on a biobank, data acquisition would require collecting all relevant corresponding images from the local picture and archiving communication system (PACS). Subsequently, curation may require selection of the appropriate sequences, vascular phases, and imaging planes. This step may also require excluding outlier cases due to imaging artifacts.

### Data exploration and quality control

Data *exploration* step consists in assessing general qualitative (e.g., through visualization) or quantitative properties (e.g., through statistics) of the initial raw dataset, in order to exhibit specific features, global trends, or outliers.

### Data labeling

Radiologists typically perform measurements, draw regions of interest, and comment images with annotations. *Markup* refers to “graphic symbols placed on an image to depict an annotation,” whereas *annotation* refers to explanatory or descriptive information regarding the meaning of an image that is generated by a human observer [49].

After selection of appropriate images, data labeling may require delineating lesions, either through bounding boxes or segmentation masks accompanied by annotations on the type of lesions and their location. Different tools can be used for image processing such as MITK Workbench [50]. Every lesion must be segmented, annotated, and, if possible, properly tracked over time on various examinations.

Markups can vary depending on the intent of a project. For example, bounding boxes may be sufficient for a detection task, whereas pixel-wise contours may be required for segmentation tasks. Further, the level of annotation details may also vary depending on the scope of a project. For example, annotation of lesion type (e.g., cyst, hemangioma, metastasis) would be required for classification tasks and consistent lesion identification (e.g., patient number, lesion number, lobe, segment) would be required for monitoring tasks.

### Reference standard

The reference standard, also known as “ground truth,” represents the knowledge that the model is expected to learn. Such reference standard may vary depending on the task, consisting in bounding boxes for detection, pixel-wise contours for segmentation, annotations for classification, measurement markups for monitoring, and clinical outcomes for prediction or prognosis.

Depending on the project, the choice of reference standard may include (1) histopathology, (2) follow-up examinations, (3) alternative imaging modality (e.g., magnetic resonance imaging [MRI]), or (4) clinical outcomes (e.g., time to tumor recurrence, disease-specific survival).

When human observation or expertise is required to establish the reference standard, additional considerations may apply such as the need for a single vs. multiple readers, the reliance on weak (novice or natural language processing on written reports) vs. strong (expert) labelers, and the adjudication process for defining the ground truth.

## Types of learning

**Fig. 4:**
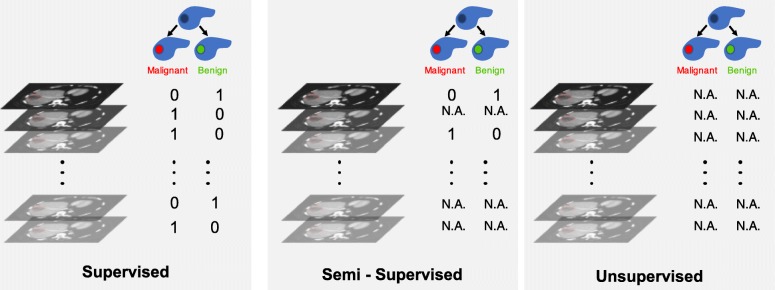
Types of learning. With supervised learning, the number of inputs (CT images in this example) equals numbers of targets (malignancy status of a lesion here). With semi-supervised, the number of inputs is greater than the number of targets (dataset includes unlabeled samples). With unsupervised learning, none of the inputs are labeled (e.g*.*, clustering, manifold learning, restricted Boltzmann machines). N.A. indicates not available information

Figure 4 illustrates the types of learning: supervised, semi-supervised, and unsupervised learning.

For *supervised learning*, a reference standard must be available for all cases. For *semi-supervised learning*, a reference standard is available only for a subset of subjects. Semi-supervised learning that relies on a combination of labeled and unlabeled data generally achieves better results than supervised learning that relies on the subset of labeled data only [51]. This learning process combines unsupervised and supervised techniques. For *unsupervised learning*, a reference standard is unavailable. In this context, unsupervised algorithms are intended to establish an efficient representation of the initial dataset (e.g., clustering through dataset statistical properties, densities, or distances) [52–54]. Such new representation may constitute an initial step before training supervised model, allowing improved performances.

Building large medical datasets of high quality is challenging and costly, due to resources required for data collection and expert time for annotation. To address these limitations, some specific training strategies or models architectures have been proposed, such as weak labeling [55, 56] or few shots learning [57].

**Fig. 5:**
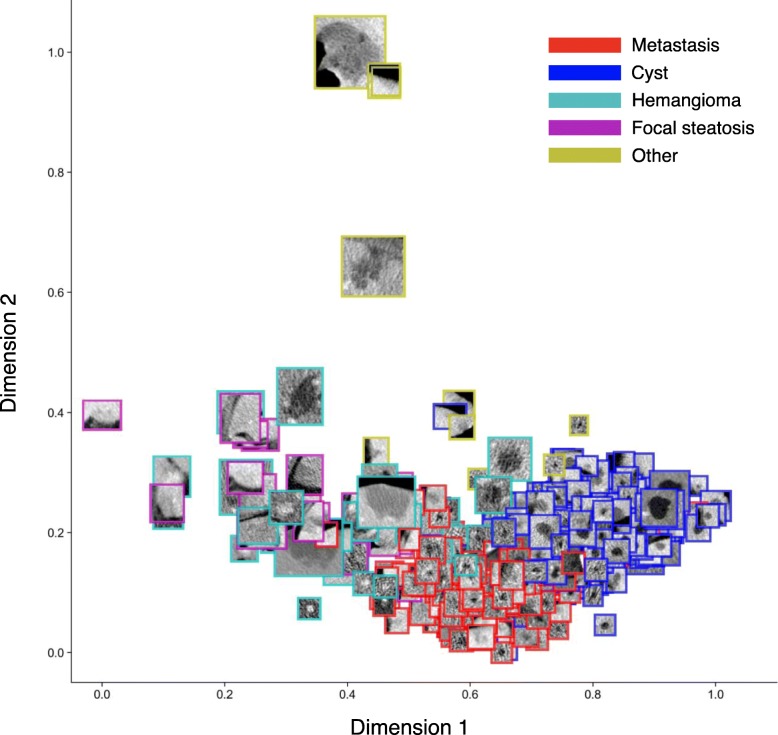
Example of data visualization: projection on first two dimensions of linear discriminant analysis (LDA) applied to radiomics features extracted from various types of lesions [58]

Once the steps described above are completed, visualization of the dataset based on extraction of radiomics features [13] and with appropriate labels can be performed prior to training with deep learning models (Fig. 5).

## Dataset sampling strategies

Data sampling refers to selection of subsets of data for training purpose. The ability of an algorithm to perform a specific task on unseen data is called generalization. To optimize and measure this performance, the entire available dataset needs to be divided in different sets. The samples in all sets should share the same data-generating process, while being independent from each other and identically distributed.

**Fig. 6:**
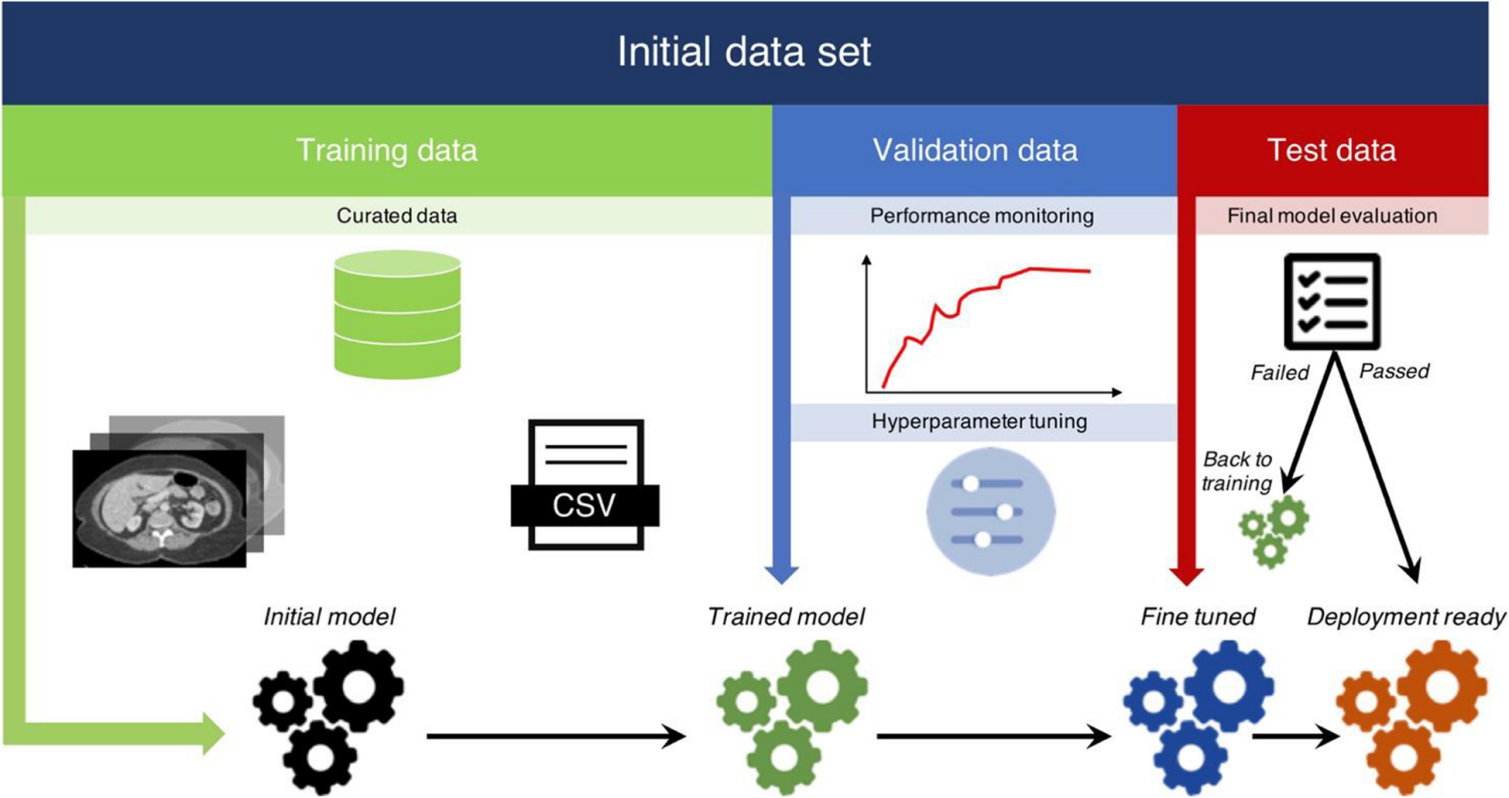
Division of dataset into training, validation, and test datasets. It is recommended to perform splitting at the very beginning of the workflow, keeping test data unseen to the model until final performance evaluation

The most frequent sampling strategy in deep learning is to divide the dataset in training, validation, and test sets (Fig. 6). The optimal ratio of samples distributed in each set varies for each problem. But as a rule of thumb, a split of 80% training, 10% validation, and 10% test division is commonly used. This division allows multiple trainings using the same training set to search for the optimal hyperparameters to maximize performance on the validation set. When the best performance is obtained on the validation set, the algorithm is ultimately used once on the test set to measure and confirm the final performance.

**Fig. 7:**
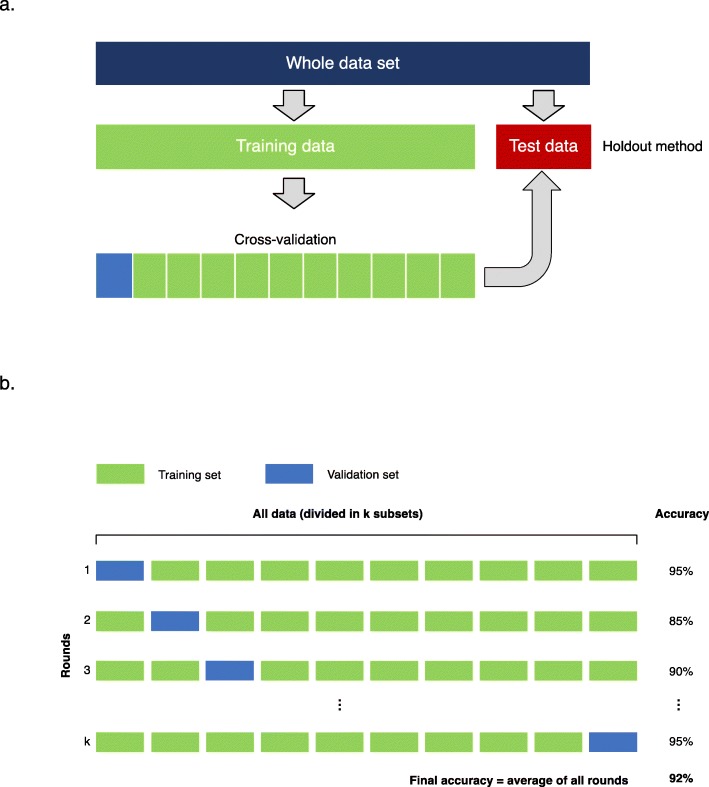
Data sampling strategies. **a** The whole dataset is split in two distinct subsets for training and testing purposes. Training dataset is subdivided to perform cross-validation, (**b**) k-fold cross-validation. The training dataset is divided in k subsets of equal size. Training is performed sequentially, considering at each iteration a specific subset as validation set

For smaller datasets, the most commonly used sampling strategy is the *k*-fold *cross-validation* [59]. The dataset is divided equally in *k* folds. For each training, the algorithm is trained on almost all folds but tested on a single holdout fold of the data. The training is repeated *k* times using varying holdout folds. The final performance is the mean of the *k* measured performances (Fig. 7).

Deep learning algorithms generally introduce two significant limitations to systematically use k-fold cross-validation. First, training deep learning algorithms on large datasets usually implies an intensive computational burden which prevents in practice a high number of training iterations with limited resources. Second, training of deep neural networks depends on many more hyperparameters than shallower machine learning algorithms.

## Deep learning libraries and architectures

**Fig. 8:**
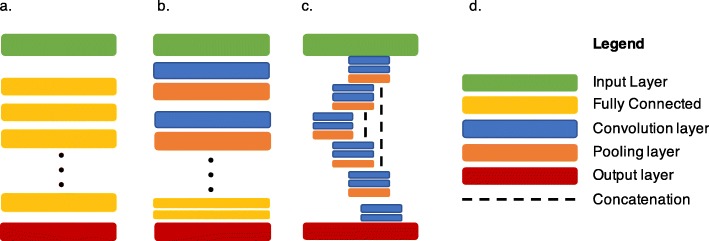
Commonly used deep learning architectures. **a** Fully connected neural networks. **b** Convolutional neural networks for detection or classification. **c** U-net, for segmentation. **d** Legend illustrating the building blocks in various deep learning architectures

Figure 8 illustrates the architecture of various deep neural networks used in medical imaging.

Deep learning methods encapsulate many levels of mathematical concepts, mostly based on linear algebra, calculus, probability, and numerical computation. Conceptually, deep learning libraries allow a higher level of programming interface to define and train deep neural networks and efficiently use available computational resources like the GPU or CPU [60].

Several open-source libraries are available with variable permissive licenses [61]. For research, the most commonly used libraries in 2019 are Tensorflow [62] and PyTorch [63]. Keras [64], Fastai, and Lasagne [65] are high-level neural network application interfaces running on top of Tensorflow, Pytorch, or Theano, respectively. Globally, Python is currently the most frequently used programming language for deep learning [62–64]. However, libraries such as Tensorflow or Caffe [66] provide alternatives supporting C++ and Matlab [67].

All of these libraries allow the implementation of the frequently used neural network architectures designed for the specific tasks above-mentioned. The deep convolutional neural network (CNN) is the architecture that enabled most of the recent advances in computer vision and medical imaging since 2012 [68]. More specifically, the convolutional layer is the basic building block used in most of the specialized architectures reporting state-of-the-art performance for classification, detection, and segmentation tasks in a wide variety of applications [3, 69]. CNN are more frequently trained on 2D images. CNN can also be trained on 3D volume of images, generated by cross-sectional abdominal imaging like computed tomography (CT) or MRI, but is a larger burden computationally. Neural network architectures evolve rapidly and the choice of network or model to use vary depending on the intended tasks. State-of-the-art results are currently achieved with architectures such ResNet and DenseNet for application such as classification and U-nets for segmentation.

Recurrent neural networks are targeted on sequential data like text or speech [70]. They are frequently used for natural language processing to extract categorical labels from radiology reports. In abdominal imaging, multiple cross-sectional follow-up exams or an ultrasound cinematic series are examples that can partly be considered as sequential.

Deep neural networks can be trained with random initialization of the internal weights or with more evolved strategies such as Glorot initialization [58]. In transfer learning, the network weights are initialized from a previous training on a different dataset. Effectiveness of transfer learning depends mostly on the similarity and complexity of the data and trained task between the previous and current datasets [71].

## Performance metrics

When training an algorithm for a research project for clinical practice, it is critical to clearly understand the metrics used to evaluate the task performance. Specific metrics are defined for each computer vision task, which may differ from a clinical objective.

The classification task is closely related to the common radiological interpretative task of providing a diagnosis from images. Consequently, for this task, the machine learning metrics are very similar to the usual diagnostic test metrics reported in diagnostic radiology. A confusion matrix defines true/false positives and true/false negatives by comparing the algorithm output values with the ground truth values. Accuracy, sensitivity, specificity, precision, and recall can then be inferred. F1 score combines precision and sensitivity. All of these performance metrics are calculated using a fixed classification threshold.

The receiver operating characteristic (ROC) curve illustrates the diagnostic performance at various classification thresholds. The area under the ROC curve (AUC) is frequently used to compare different algorithms on the same task. To select only a clinically useful range of operation, partial AUC can also be used [72].

Detection and segmentation tasks frequently use interchangeable metrics. The purpose is to evaluate quantitatively the similarity of an automatically generated bounding box or a segmentation mask to the associated ground truth defined by an expert radiologist. Intersection over union (IOU) is defined by the area delimited by the intersection of two bounding boxes divided by the union of the same two bounding boxes. For segmentation, the Dice or Jaccard coefficients are also a similarity metrics at the pixel level that can directly be calculated from IOU.

Table 2 summarizes reference standards, performance metrics, and model selection for various tasks.

**Table 2:** Examples of reference standards, common performance metrics, and model selection for various tasks

	Detection	Segmentation	Classification	Prediction
Features	-Bounding boxes -Masks	-Lesion patch -Full image at max diameter -Radiomics features -Masks	-Lesion patch -Radiomic features	-Lesion patch -Time to recurrence -Survival time -TRG
Model architectures	-CNN	-U-Net	-Fully connected	-CNN
Performance metrics	-Intersection over union (IOU) -Mean average precision (mAP)	-Dice score -IOU	-Receiver operating characteristic (ROC) -Accuracy	-ROC curve -Accuracy -*R*^2^

## Hardware

Hardware selection refers to determining the technical specifications based on a given deep learning model. Key parameters to consider when selecting hardware are dataset volume and model complexity. Deep learning models can be trained on CPU, GPUs, or on cloud computing platforms, which may leverage deep learning-oriented devices such as tensor processing units (TPU). Briefly, CPUs are of interest for sequential calculations and take advantage of a large available memory but suffer from limited memory bandwidth. In contrast, GPUs and TPUs are architectures of choice for massive parallel computing, offering limited memory size but at very high bandwidth. Larger GPU memory facilitates training of deeper models with a higher number of trainable parameters. Commercial GPUs currently offer memory size between 8 and 32 GB allowing training of most recent CNN architectures at sufficient image resolution for medical imaging. Considering the high computational cost related to model training, especially when considering large datasets of images with CNNs, GPU are generally preferred [73]. Multi GPU is a good way to increase computational performance on local stations, but such configurations generally imply additional hardware considerations (e.g*.*, power supply and cooling). Each hardware solution exhibits specific architectures, memory types, volumes, and associated bandwidths. Training performances of typical deep learning models (e.g*.*, fully connected, recurrent, convolutional) can vary drastically from one platform to another [74].

When training time is a key parameter for large datasets, cloud computing solutions can be advantageous. Cloud computing refers to internet-based services using a third-party hardware resources leveraging large storage and technical resources. In this field, Microsoft Azure, Google Cloud platform, and Amazon AWS are major stakeholders. Each of these platforms exhibits specific accessible hardware, services, and fares. Main advantages of cloud computing platforms are the easy access to high computational power, almost unlimited storage, cost-efficiency, and low maintenance.

However, cloud computing solutions suffer from specific shortcomings such as technical issues (data are fractioned and stored at multiple locations; thus, one server off can cause subsequent issues). Additionally, transferring datasets to remote servers lead inevitably to data security and integrity questions depending on server location, including possible attacks. It is thus important to first check security procedures proposed by each solution provider and to ensure that no sensitive information are transferred on remote servers. In this context, de-identification and patient anonymization concepts as presented above are of paramount importance [75].

## Implementation and practical considerations

Implementation refers here to executing a previously established designed deep learning project. In the current context, it encompasses building and curing the dataset [76], choosing a set of neural networks architectures, training the networks and fine tuning hyperparameters using selected metrics.

Deployment refers to the implementation of a locally developed solution to a larger scale, such as at the institution level or within a healthcare network. This process requires clear definition of model specifications, in terms of performance (e.g*.*, optimizing sensitivity or specificity based on ROC curves) or software engineering (e.g*.*, configurations, versioning, unit-testing, or specific institutional requirements).

It is recommended to regularly monitor model performances to detect any potential bias or accuracy loss. Depending on the evolution of performance metrics and visual assessment over time, a model may be retrained using additional data to dynamically update its performance. Additionally, it is recommended to store weights obtained after training separately from network architecture at regular checkpoints. This allows easier updates and versioning, as long as network architecture remains identical.

From a practical perspective, integration of models into routine procedures can be challenging in terms of portability, data accessibility, and preprocessing. It is thus necessary to define if developed solution is intended to be integrated into an existing infrastructure or used as a standalone application. During first phase of deployment, a containerized approach such as that proposed by Docker [77, 78] or Kubernetes [79] may be adopted and web-based applications (REST-API) for subsequent deployment.

To better fulfill these integration challenges, marketplaces of AI applications are rapidly emerging offering a wide variety of tools with a unified user interface for radiologists and a generic application programming interface (API) for developers. This commercial layer between PACS vendors and AI applications can potentially allow faster clinical deployment, validation, and usability.

## Regulatory framework

A commercial software dedicated to medical imaging is generally recognized by most regulating jurisdictions as a medical device and more specifically as a software as a medical device (SaMD) using the proposed International Medical Device Regulators Forum (IMDRF) terminology [80]. This international framework categorizes the associated risk based on the intended medical purpose and the targeted healthcare situation to better determine the needed pathway of regulation. Conceptually, an application diagnosing a critical condition will need a more rigorous and extensive regulation process than an application that inform clinical management for a non-serious condition [81]. Depending on the risk categorization, the software must satisfy criteria for a quality management system (QMS) and for clinical evaluation. Based on these building blocks, each regulatory jurisdiction implements its own regulatory pathways. Most jurisdictions also follow the ISO – IEC 62304:2006 - Software Life Cycle Processes framework in their implementation [82].

To cover the specific challenges of SaMD trained on patient data using deep learning algorithms, namely AI/ML-based SaMD, many jurisdictions are currently reviewing their regulatory frameworks to reflect the evolutionary aspect of these applications [83]. Of note, the capacity to rapidly retrain models on new available data to improve performance or even to change the intended use is a new software paradigm that needs regulatory update.

## Conclusion

Deep learning shows great promise in radiology, as demonstrated by the diversity of applications [10] and reported performances in a variety of computer vision tasks [3].

This paper provided an overview of the steps to undertake a deep learning project in radiology, from task definition to deployment, and scaling. As medical applications are numerous and technical solutions are easily accessible, the most time-consuming part is dataset building (data collection and curation of structured or unstructured data), followed by model fine tuning through hyperparameters optimization.

On a multi-institutional scale, the large amount of available shared data constitutes a great opportunity for complex model training. The main limitations are the availability of expert annotations, the pooling of data across multiple sites and the need for data curation to achieve a high-quality dataset. To overcome privacy concerns about data breach, a potential solution may be to perform local training of multiple models and to share the weights, a strategy known as federated or distributed learning.

## Data Availability

Data sharing is not applicable to this article as no datasets were generated or analyzed during the current study.

## Abbreviations

AI: Artificial intelligence
API: Application programming interface
AUC: Area under curve
CAD: Computer-aided diagnosis
CNN: Convolutional neural network
CPU: Central processing unit
CT: Computed tomography
DICOM: Digital imaging and communications in medicine
DL: Deep learning
GPU: Graphical processing unit
IMDRF: International Medical Device Regulators Forum
IOU: Intersection over union
IRB: Institutional review board
ML: Machine learning
MRI: Magnetic resonance imaging
PACS: Picture and archiving communication system
QMS: Quality management system
ROC: Receiver operating characteristic
SaMD: Software as a medical device
TPU: Tensor processing unit
TRG: Tumor response grade
